# Acceptance, Needs, and Demands for Nutritional mHealth Support in Patients with Cardiovascular Disease

**DOI:** 10.3390/nu16234155

**Published:** 2024-11-30

**Authors:** Darya Mohajeri, Lisa Maria Jahre, Alexander Bäuerle, Theresa Schieffers, Daniel Messiha, Christos Rammos, Martin Teufel, Tienush Rassaf, Julia Lortz

**Affiliations:** 1 Department of Cardiology and Vascular Medicine, West-German Heart and Vascular Center Essen, University of Duisburg-Essen, Hufelandstr. 55, 45147 Essen, Germany; darya.mohajeri@uk-essen.de (D.M.); t.schieffers@web.de (T.S.); daniel.messiha@uk-essen.de (D.M.); christos.rammos@uk-essen.de (C.R.); tienush.rassaf@uk-essen.de (T.R.); 2 Clinic for Psychosomatic Medicine and Psychotherapy, LVR-University Hospital Essen, University of Duisburg-Essen, Virchowstr. 174, 45147 Essen, Germany; lisa.jahre@lvr.de (L.M.J.); alexander.baeuerle@lvr.de (A.B.); martin.teufel@lvr.de (M.T.); 3 Center for Translational Neuro- and Behavioral Sciences (C-TNBS), University of Duisburg-Essen, Virchowstr. 174, 45147 Essen, Germany

**Keywords:** acceptance, cardiovascular disease, eHealth, mHealth, nutrional support, secondary prevention, UTAUT

## Abstract

Background: Cardiovascular diseases (CVDs) are the leading causes of death globally. Managing risk factors and preventing atherosclerosis and its progress, especially with lifestyle changes, are highly important. Smartphone-based mobile health (mHealth) strategies allow easily accessible assistance for healthy nutrition. This study aimed to assess the acceptance and outline the needs and demands for a nutritional mHealth tool by analyzing the desired characteristics. Methods: A cross-sectional study was conducted between August 2022 and September 2023 targeting 398 individuals with atherosclerosis. Acceptance, needs, and demands regarding mHealth, sociodemographic, medical, psychometric, and electronic health (eHealth) data were assessed. Multiple hierarchical regression analyses were conducted to determine the predictors of acceptance. Results: High acceptance for nutritional mHealth was reported by 88.4% (*n* = 274). Significant predictors of acceptance were age (β = −0.01, *p* = 0.002), diabetes (β = 0.20, *p* = 0.041), depressive symptoms (β = −0.02, *p* = 0.017), digital confidence (β = 0.17, *p* = 0.001), Internet anxiety (β = −0.18, *p* = 0.004), and the Unified Theory of Acceptance and Use of Technology (UTAUT) predictors effort expectancy (β = 0.23, *p* < 0.001) and social influence (β = 0.53, *p* < 0.001). Preferences included handheld devices, permanent use (86.5%), and weekly (44.5%) new content of 10 to 30 min (79%). Conclusions: These results summarize the patients’ preferences for individualized mHealth tools to ensure their effectiveness. Especially regarding the secondary prevention of CVDs, mHealth can be a helpful resource. The high acceptance rate and specific preferences outlined in this study form a strong basis for the development of mHealth tools with a focus on nutritional support in patients with CVDs.

## 1. Background

The World Health Organization (WHO) still identifies cardiovascular disease (CVD) as the leading cause of mortality worldwide [1]. Arterial hypertension, hypercholesterolemia, hyperglycemia, and obesity, often stemming from an unhealthy lifestyle, are common cardiovascular risk factors that contribute to the progression of atherosclerosis, the primary mechanism behind CVD development [2]. Managing these risk factors through both medical interventions and lifestyle changes can significantly reduce disease progression and prevent deaths related to CVD [3,4,5].

As a modifiable risk factor, nutrition plays a vital role in CVD prevention by targeting dyslipidemia, overweight, and hyperglycemia. Various dietary recommendations, such as the Mediterranean diet, have been shown to be protective in both primary and secondary prevention [6,7]. However, since the reported adherence to general lifestyle modification in secondary prevention is only 4.3% [8], a sustainable implementation of behavioral change is lacking. As the prevalence of nutrition-related medical conditions continues to rise despite numerous disease-focused initiatives, the integration of new approaches, such as eHealth technologies, becomes increasingly crucial. These technologies play a pivotal role in promoting health awareness and facilitating the adoption and maintenance of healthy lifestyle habits while also managing stress [9,10,11]. Their seamless integration into routine healthcare practices stands to profoundly benefit both individuals and healthcare providers [12,13,14].

While traditional nutritional management and health improvement programs emphasizing balanced nutrition and physical activity have been proven to be effective [15,16], face-to-face interventions are accompanied by labor-intensive, expensive, and resource-heavy limitations. Furthermore, comprehensive nutritional management programs typically necessitate more than a year of sustained support through in-person interactions [17], thus requiring novel digital therapeutic strategies.

Electronic health (eHealth) interventions have demonstrated a significant positive impact on health outcomes [18,19,20]. Particularly in the realm of CVD risk reduction, their widespread adoption has faced notable barriers, especially among older individuals [21]. However, challenges such as affordability, usability, and concerns regarding privacy and security persist [22,23]. Reports indicate poor adherence to eHealth interventions targeting CVD risk reduction, with high refusal rates among heart failure patients for telehealth usage due to similar barriers [24]. One subgroup of eHealth consists of mobile health (mHealth), which includes mobile devices for digital health [20]. 

Addressing these barriers is paramount in the development of future eHealth interventions. In recent years, there has been a growing recognition of the importance of user-centered design approaches in the realm of digital health innovations [25,26,27,28]. However, a systematic review highlighted that patients are often engaged in the development process at a late stage, limiting their ability to meaningfully influence content and design decisions [29]. Achieving patient-centeredness requires the early identification and consideration of the patients’ needs and interests in the development process.

The Unified Theory of Acceptance and Use of Technology (UTAUT) provides a framework for understanding the patients’ acceptance regarding technology including factors such as performance expectancy (PE), effort expectancy (EE), and social influence (SI), all three of which predict the overall behavioral intention (BI) [18,30]. Continuous advancements in mHealth therapeutic approaches are necessary to meet the evolving needs and demands of patients with CVD. Therefore, this study aimed to identify specific characteristics highly valued in mHealth tools for this patient population, ensuring their effectiveness and acceptance in clinical practice. 

## 2. Objectives

This study sought to investigate the acceptance of mHealth tools for nutritional support as well as its underlying factors. Furthermore, the specific attributes for an mHealth intervention designed for secondary prevention, particularly focusing on nutritional support for CVD management desired by patients with CVDs, are outlined.

## 3. Methods

### 3.1. Study Design and Study Population

A cross-sectional observational study was conducted to investigate the acceptance, needs, and demands of nutritional mHealth support among individuals with atherosclerosis. Participants were mostly recruited in person at the Department of Cardiology, West German Heart and Vascular Center, University Hospital Essen. A small percentage was also recruited via newspaper and online media. Participation was anonymous and voluntary, with no compensation offered. Patients were informed on data protection both verbally and written and had to give their consent to begin the survey. Inclusion criteria were self-reported diagnosis of a cardiovascular disease, age, sufficient command of the German language, and Internet access.

Patients were excluded if they did not agree with the written informed consent or did not have an atherosclerotic disease. The absence of a mobile tool such as a tablet or mobile phone and the absence of Internet access also led to exclusion. Children were not surveyed. Data were collected between August 2022 and September 2023 via an online questionnaire on the platform Unipark [31]. At the beginning of the survey, the study information was presented, and digital informed consent was given by participants before the survey started. On average, it took M = 21 (SD = 10) min to complete the survey. The questionnaire was initially started by *n* = 398 participants. Due to not fulfilling the inclusion criteria or missing data on the primary outcome (acceptance), *n* = 88 (22.1%) participants had to be excluded. Therefore, *n* = 310 (77.9%) participants were included in the final data analysis. The study was approved by the Ethics Committee of the Medical Faculty of the University of Duisburg-Essen (19-89-47-BO).

### 3.2. Assessment Instruments

The survey consisted of sociodemographic, medical, and eHealth data. Furthermore, the needs and demands regarding nutritional mHealth support for atherosclerosis were assessed. The primary outcome was acceptance, operationalized as behavioral intention (BI). Validated assessment instruments and established self-generated items were used to collect the responses.

Sociodemographic data included age, gender, marital status, educational level, occupational status, and place of residence (population size).

Medical data consisted of body weight (in kg) and height (in cm). Participants were asked whether they had experienced a heart attack or underwent coronary stent or bypass surgery. Furthermore, the participants responded whether they were prescribed cholesterol-lowering medication and if they were active smokers. Comorbid diseases (e.g., arterial hypertension, hyperlipidemia) and feeling of tightness during physical exertion were assessed. The participants indicated how many flights of stairs they were able to climb without shortness of breath (‘0’ to ‘no restrictions’) and stated the walking distance they were able to manage (e.g., ‘I have no restrictions and can manage longer distances without discomfort (no pain)’). Moreover, the participants gave responses regarding their physical activity (‘e.g., once per week or less’) and reported whether they had been diagnosed with a mental illness.

Depressive symptoms were assessed with the Patient Health Questionnaire-8 (PHQ-8) [32]. The questionnaire consists of eight items and responses are given on a four-point Likert scale (0 = ‘never’, 3 = ‘almost every day’). Sum scores ≥ 10 indicate severe depressive symptoms [32]. In this sample, the internal consistency was high (Cronbach’s α = 0.80).

Digital confidence [18,33,34] was assessed via three items (e.g., ‘How confident are you in using digital media?’). Responses were given on a five-point Likert scale (1 = ‘not very confident’, 5 = ‘very confident’). Internal consistency for digital confidence was high (Cronbach’s α = 0.96). Furthermore, Internet anxiety [34,35,36] (e.g., ‘I have concerns about using the Internet.’) and digital overload [18,36,37] (e.g., ‘I feel burdened by the constant accessibility via cell phone or e-mail.’) were assessed via three items each. Responses were given on a five-point Likert scale (1 = ‘strongly disagree’, 5 = ‘strongly agree’). Internal consistency was good (Cronbach’s α = 0.90 for Internet anxiety, α = 0.86 for digital overload).

The validated UTAUT model [38] was applied to examine acceptance toward nutritional mHealth support for atherosclerosis and its underlying predictors. Acceptance was operationalized as behavioral intention (BI) and assessed by four items (e.g., ‘I would use such mHealth strategies if they were offered to me.’). Internal consistency for BI was high (Cronbach’s α = 0.93). Social influence (SI) was determined via three items (e.g., ‘People close to me would approve of the use of such mHealth strategies’). Internal consistency for SI was high (Cronbach’s α = 0.90). Four items were used to assess the performance expectancy (PE, e.g., ‘Such mHealth strategies could improve my overall well-being’). Internal consistency for PE was high (Cronbach’s α = 0.93). Effort expectancy (EE) was examined with two items (e.g., ‘Using such mHealth strategies would not be an additional burden for me’). Internal consistency for EE was good (Cronbach’s α = 0.74). Responses were given on a five-point Likert scale (1 = ‘strongly disagree’, 5 = ‘strongly agree’).

Needs and demands regarding nutritional mHealth support for atherosclerosis were assessed via different items including dichotomous and Likert-scaled assessments. The self-generated items were well-established in previous studies [10,27,39].

Participants responded to items regarding the availability of mHealth strategies (e.g., ‘smartphone’, ‘tablet’), format of the content (e.g., ‘app’, ‘audio or video material’), duration of use (e.g., ‘1 to 10 min’, ‘over 45 min’), frequency of new content (e.g., ‘daily’, ‘weekly’), and session length (e.g., ‘1 to 10 min’, ‘>45 min’). Moreover, the participants rated different topics that should be addressed by nutritional mHealth support for atherosclerosis. The list of topics was compiled by experts from the field of psycho-cardiology. Responses were given on a five-point Likert scale (0 = ‘not relevant’, 4 = ‘very relevant’).

### 3.3. Statistical Analysis

Statistical analysis was performed using R (4.3.1). Mean scores were calculated for digital confidence, digital overload, Internet anxiety, and for acceptance (operationalized as BI) as well as its three predictors (EE, PE, SI). For PHQ-8, the sum scores were calculated. The body mass index (BMI) was calculated based on body weight and height (kg/m^2^). Acceptance was divided into three categories, in accordance with prior research [18,34,40] for descriptive purposes (multiple hierarchical regression analysis were conducted with the mean score of acceptance): low acceptance (scores from 1 to 2.34), moderate acceptance (2.35 to 3.67), and high acceptance (3.68 to 5). Descriptive statistics were computed in the form of distributions, mean scores, and standard deviations for acceptance and its predictors, needs, and demands, and further descriptive data. To examine the drivers and barriers of acceptance of nutritional mHealth support for atherosclerosis, a multiple hierarchical regression analysis was conducted. Predictors were included block wise: (1) sociodemographic data, (2) medical data, (3) eHealth data, and (4) UTAUT predictors. Absence of multicollinearity was examined with the variance inflation factor (VIF). All VIF values were ≤4.5. A normal distribution of the residuals was assumed because visual inspection of the qq-plots of the residuals showed no signs of violations against normality. Homoscedasticity was verified via scatter plots of the standardized residuals and the adjusted predicted values. The level of significance was set to α < 0.05 for all tests. Effect sizes were reported according to Cohen (1988) [41], with values around 0.2, 0.5, and 0.8 indicating small, medium, and large effects, respectively.

## 4. Results

### 4.1. Study Population

Of the *n* = 310 individuals with atherosclerosis, 58.1% (*n* = 180) were male. On average, participants were M = 61.72 (SD = 11.44) years old, ranging from 34 to 84 years. The average BMI was M = 27.67 (SD = 5.94) kg/m^2^. One third of the participants reported normal weight (36.5%, *n* = 113) and one third overweight (pre-obesity, 33.2%, *n* = 103). Among the study sample, 26.5% (*n* = 82) had a myocardial infarction in the past and 41.0% (*n* = 127) underwent coronary stenting or bypass surgery. The majority (69.0%, *n* = 214) were prescribed cholesterol-lowering medication. One third (31.9%, *n* = 99) of the sample were active smokers.

A diagnosis of mental illness was reported by 21.3% (*n* = 66). The average PHQ-8 score was M = 4.63 (SD = 4.01) with 11.6% (*n* = 36) of the sample reaching the cut-off score indicating major depressive symptoms. Digital confidence was high (M = 3.87, SD = 0.98, range 1–5), while the digital overload (M = 1.94, SD = 1.01, range 1–5) and Internet anxiety (M = 1.58, SD = 0.80, range 1–5) were low. Table 1 includes a further description of the study sample.

### 4.2. Acceptance of Nutritional mHealth Support in Patients with an Atherosclerotic Disease

Overall, the acceptance of nutritional mHealth support for atherosclerosis was high (M = 4.35, SD = 0.69, range 1–5). Most of the participants reported high acceptance (88.4%, *n* = 274), and 10.3% (*n* = 32) showed moderate acceptance, while only 1.3% (*n* = 4) of participants reported a low acceptance of nutritional mHealth support.

### 4.3. Predictors of Acceptance of Nutritional mHealth Support in Patients with an Atherosclerotic Disease

Multiple hierarchical regression analysis was applied to determine the predictors of the acceptance of nutritional mHealth support for atherosclerosis.

In the first step, sociodemographic data were included (R^2^ = 0.046, R^2^_adj_ = 0.030, F(5, 304) = 2.90, *p* = 0.014). *Age* (β = −0.18, *p* = 0.002) was a significant predictor of acceptance. The explained variance of the first step was 4.6%.

Medical data were included in the second step (R^2^ = 0.083, R^2^_adj_ = 0.052, F(10, 299) = 2.69, *p* = 0.004). The explained variance significantly increased to 8.3% (∆R^2^ = 0.037, F(5, 299) = 8.07, *p* < 0.001). Diabetes mellitus (β = 0.29, *p* = 0.041) and depressive symptoms (β = −0.14, *p* = 0.017) were significant predictors of acceptance.

eHealth data were included in the third step (R^2^ = 0.190, R^2^_adj_ = 0.155, F(13, 296) = 5.35, *p* < 0.001), which significantly increased the explained variance to 19.0% (∆R^2^ = 0.107, F(3, 296) = 39.16, *p* < 0.001). Digital confidence (β = 0.25, *p* < 0.001) and Internet anxiety (β = −0.20, *p* = 0.004) were significant predictors of acceptance.

In the final step, the three UTAUT predictors were included (R^2^ = 0.731, R^2^_adj_ = 0.717, F(16, 293) = 49.84, *p* < 0.001). Explained variance of the final model was significantly increased to 73.1% (∆R^2^ = 0.541, F(3, 293) = 196.66, *p* < 0.001). *SI* (β = 0.52, *p* < 0.001) and *EE* (β = 0.27, *p* < 0.001) were significant predictors. Additionally, place of residence: rural area (β = 0.94, *p* = 0.004) was a significant predictor of acceptance in the final step. Table 2 contains the final UTAUT model of acceptance and its predictors.

### 4.4. Needs and Demands Regarding mHealth Strategies for Nutritional Support in Patients with an Atherosclerotic Disease

Patients with atherosclerotic disease reported a variety of needs and demands regarding nutritional mHealth support, which are shown in Table 3. Availability on a smartphone had the highest approval (M = 4.67, SD = 0.96) compared to other devices. In the same way, the most preferred format was an app (M = 4.57, SD = 1.06). Most of the participants (86.5%, *n* = 268) reported that they would use an app permanently. Nearly half of the sample (44.5%, *n* = 138) stated that new content should be added weekly. The preferred session length was 10 to 20 min (40.0%, *n* = 124) and 20 to 30 min (39.0%, *n* = 121).

### 4.5. Relevant and Irrelevant Content for Nutritional mHealth Support in Patients with an Atherosclerotic Disease

**Figure 1:**
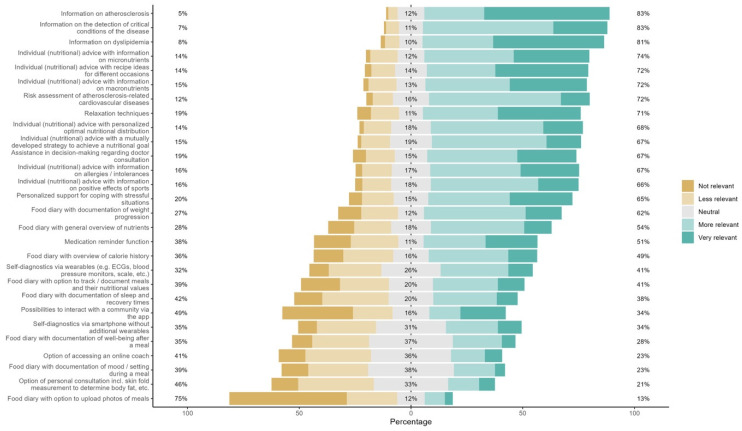
Relevant and irrelevant content for nutritional mHealth support in patients with an atherosclerotic disease.

The most relevant topics for nutritional mHealth support were educationally oriented as topics such as ‘Information on atherosclerosis’ (83%), ‘Information on the detection of critical conditions of the disease’ (83%), and ‘Information on dyslipidemia’ (81%) were prioritized. The topics ‘Food diary with option to upload photos of meals’ (13%), ‘Option of personal consultation including skin fold measurement to determine body fat, etc.’ (21%), ‘Food diary with documentation of mood/setting during a meal’ (23%), and ‘Option of accessing an online coach’ (23%) were considered the least relevant. A detailed overview of all responses is presented in Figure 1.

## 5. Discussion

This study investigated the acceptance and preferences for mHealth interventions focused on nutritional support among individuals with atherosclerosis. The study population was primarily male, with an average age of approximately 62 years. The mean BMI was categorized as overweight, reflecting a representative population with CVD [6]. The sample included a significant proportion of individuals who had experienced myocardial infarction and those who had undergone coronary stenting or bypass surgery, also reflecting a population at high risk for recurrent cardiovascular events, underscoring the need for effective secondary prevention strategies including nutritional management.

Individuals with atherosclerosis stated a high overall acceptance of nutritional mHealth strategies, with almost 90% of participants reporting high acceptance and only 1.3% indicating low acceptance. This high level of acceptance suggests that mHealth tools, if designed appropriately, have the potential to be well-received by this population. The high acceptance rate aligns with previous studies indicating that mHealth interventions are generally well-received in populations with chronic diseases due to their convenience and the personalized support they can provide [12,14,19,20,21].

Especially regarding nutritional support, mHealth tools are promising for supporting the patients’ goals by either simplifying weight loss or by facilitating optimized secondary prevention (e.g., via a reduction in low-density lipoprotein (LDL) and HbA1c levels). A meta-analysis conducted with 13 studies regarding the effectiveness of mHealth dietary interventions showed a significant improvement in systolic blood pressure and LDL, although the body weight and BMI did not change [12]. Another meta-analysis including 26 RCTs accentuated a significant reduction in systolic blood pressure and waist circumference [20]. This emphasizes that dietary interventions delivered via mHealth may not necessarily achieve significant weight loss but can improve the overall CVD risk profile. This finding aligns with the results of the present study, which show that dietary factors influencing the underlying disease are within the scope of the target population rather than simple weight loss tools. By addressing metabolic side effects, these interventions still have beneficial effects on long-term cardiac outcomes [6,7,8,9].

To achieve long-term benefits of these nutritional mHealth strategies, it is necessary to optimize them to meet the individual needs of the specific patient cohort. Other studies have also shown similarly high acceptance rates, with 78.3% [18] for cardiac rehabilitation and 83.4% [42] in a pilot project for an mHealth application in secondary CVD-prevention. However, a study surveying inpatient patients from different medical fields showed a far lower acceptance rate, even among CVD patients [40]. This suggests a discrepancy in the acceptance rates between inpatient and outpatient settings, possibly derived from various stress situations and different burdens of disease, which should be further investigated. Understanding the factors causing this polarity can help improve future mHealth developments.

Multiple hierarchical regression analysis revealed several significant predictors of acceptance. Sociodemographic factors and medical data as well as eHealth variables were all considered. Key predictors included younger age, diagnosis of diabetes mellitus, and lower depressive symptoms. In former studies, the existence of a mental disorder was also found to be a predictor of higher acceptance [18,36,39]. This might be explained by the fact that patients with higher levels of mental burden express a higher demand for care, which could be covered by mHealth strategies. Higher digital confidence and lower Internet anxiety were significant predictors, indicating that participants who are more comfortable and proficient with digital tools are more likely to embrace mHealth interventions. The combination of high digital confidence and low Internet anxiety has also previously been described as a significant predictor of higher acceptance in mHealth [18,30]. 

Social influence and effort expectancy were strong predictors of acceptance. Living in a rural area significantly predicted acceptance, possibly due to limited access to traditional healthcare resources, making mHealth tools a valuable alternative. The final model for UTAUT explained 73% of the variance in acceptance, highlighting the importance of considering a comprehensive range of factors when designing and implementing mHealth interventions, and is consistent with the findings from previous studies [30,34,36,39,40].

Participants expressed clear preferences for the features and formats of mHealth tools. The highest approval was for availability on smartphones, with a preference for app-based formats. The second preferred medium was a tablet, showing that mobile media are much more desired. As 90% of the worldwide population owns a smartphone, a wide cohort can be targeted [43]. Most participants indicated a willingness to use an app permanently, and there was a strong preference for weekly updates with new content. Adding new content can also be a reminder for more frequent app usage for the participants. Preferred session lengths were relatively short, indicating that interventions should be designed to fit into the daily routines of users without becoming burdensome. This seems to be a general preference, even in other contexts of the target population [10]. 

A common limiting factor for any therapeutic approach is patient adherence. It has been previously shown that health apps are typically used for an average of six months before participants either lose interest or no longer perceive benefits [44,45]. One approach to addressing this issue might be to implement frequent follow-ups to improve the apps based on user feedback and to introduce new features or individualized content. As reflected in our results, short sessions are favored, as they allow for easier integration into daily or weekly routines.

Participants prioritized educational content, specifically information on atherosclerosis, the detection of critical conditions, and dyslipidemia. These findings suggest that patients value informative resources that help them understand and manage their condition. Conversely, features such as food diaries with options to upload photos, personal consultations for body fat measurement, and mood documentation were considered less relevant. This feedback is critical for developers, indicating that while some interactive features might seem beneficial from a clinical perspective, they may not align with patient preferences or perceived needs [9,10]. Therefore, simply losing weight and achieving a specific BMI may not be the focus of the target population. In this context, it makes sense to link the positive influence of a healthy diet to the underlying atherosclerotic disease [11,12]. 

The findings of this study have several implications for the development and implementation of mHealth tools for nutritional support in patients with CVD. Incorporating user needs and demands is crucial for the successful adoption and sustained use of mHealth tools. This includes focusing on educational content, ensuring ease of use, and providing regular updates. However, developers should also consider demographic factors such as age and digital literacy when designing mHealth interventions to ensure that they are accessible and engaging for all users. The additional inclusion of features that address comorbid conditions like diabetes may enhance the relevance and utility of mHealth tools. Enhancing digital confidence and reducing Internet anxiety in the target population can further improve acceptance. With a rising digital health market, mHealth may complement current guidelines and facilitate new approaches for the secondary prevention and therapy of cardiovascular diseases, with a specialized focus on nutrition. 

## 6. Limitations

The results at hand may be limited due to the form of data collection. Patients were asked for self-reports and self-assessment, which means that their expectations and actual outcomes may differ in terms of actual mHealth usage for nutritional support. Patient age ranged from 34 to 82 years, with a mean age of 62 years, indicating that most participants were older. The needs and demands of younger adults may differ regarding nutritional mHealth opportunities. Nevertheless, age was a significant predictor of acceptance, so information on a relatively older population’s needs and demands are therefore of high relevance. Furthermore, patients with any kind of atherosclerotic disease were included; however, there might be differences in the needs and demands of patients within the subgroups of CVD, which should be investigated further in the future. Due to the assessment via self-report, the diagnosis of CVD could not be objectively confirmed.

## 7. Conclusions

In conclusion, this study underscores the importance of integrating patient preferences into the ongoing development and design of mHealth tools for nutritional support in CVD management to ensure their effectiveness and sustainability. By focusing on user-centered design and addressing the specific needs and demands of patients with atherosclerosis, mHealth interventions focusing on nutrition can play a vital role in the secondary prevention of cardiovascular diseases. The high acceptance and specific preferences identified in this study provide a strong foundation for the development and implementation of mHealth tools that can significantly improve the management of CVD through nutritional support. By taking into account the factors that influence acceptance, a sustainable implementation of mHealth can succeed. Features that promised higher satisfaction included short sessions with frequent updates, easily accessible information on cardiovascular diseases, and mobile accessibility. Innovative mHealth tools that are developed with these desired features are promising additions to traditional methods of secondary prevention, especially in patients with CVD and with regard to nutritional support.

## Figures and Tables

**Table 1:** Study population characteristics. Sample characteristics.

	N (%)
Marital status	
Single	43 (13.9)
In a relationship	26 (8.4)
Married	193 (62.3)
Divorced/separated	27 (8.7)
Widowed	21 (6.8)
Educational level	
No or lower education/other	79 (25.5)
Higher secondary education	98 (31.6)
Higher education entrance qualification	67 (21.6)
University education	64 (20.6)
NA	2 (0.6)
Occupational status	
In education (e.g., school, university)	2 (0.6)
Unemployed (e.g., job-seeking, unfit to work)	12 (3.9)
On sick leave	12 (3.9)
Part-time employed	29 (9.4)
Employed	96 (31.0)
Retired	140 (45.2)
Other	18 (5.8)
NA	1 (0.3)
Place of residence (population size)	
Large city (>100,000 residents)	287 (63.4)
Medium-sized city (>20,000 residents)	78 (17.2)
Small town (>5000 residents)	34 (7.5)
Rural area (<5000 residents)	54 (11.9)
BMI categories	
Normal weight	113 (36.5)
Obese (Class I)	56 (18.1)
Obese (Class II)	20 (6.5)
Obese (Class III)	14 (4.5)
Overweight (pre-obese)	103 (33.2)
Underweight	4 (1.3)
Comorbid diseases	
Arterial hypertension	214 (69.0)
Hyperlipidemia	152 (49.0)
Diabetes mellitus	75 (24.2)
Stroke	42 (13.5)
Chronic kidney disease	31 (10.0)
Coronary heart disease	137 (44.2)
Peripheral artery disease	89 (28.7)
Family history of cardiovascular diseases	190 (61.3)
Other comorbid disease	98 (31.6)
Feeling of tightness during physical exertion	160 (51.6)
Flights of stairs without shortness of breath	
0	30 (9.7)
1	48 (15.5)
2	92 (29.7)
3	41 (13.2)
4	26 (8.4)
No restrictions	73 (23.5)
Walking distance	
I have no restrictions and can manage longer distances without discomfort (no pain).	198 (63.9)
The pain-free walking distance is more than 200 m. Rest is then needed.	58 (18.7)
The pain-free walking distance is less than 200 m. Rest is then needed.	42 (13.5)
Even at rest, my legs hurt (pain at rest), making it difficult for me to walk the shortest distances.	6 (1.9)
I have severe pain at rest and see changes in my feet/legs.	6 (1.9)
Physical activity	
Once per week or less	153 (49.4)
About twice a week	58 (18.7)
About three times a week	60 (19.4)
About four to six times a week	28 (9.0)
Daily	11 (3.5)
Total	310 (100.0)

Note. NA = data not available. BMI = body mass index.

**Table 2:** UTAUT model of acceptance and its predictors. Hierarchical regression model of acceptance of mHealth strategies for atherosclerosis.

Predictors	B	β	t	R^2^	∆R^2^	*p*
(Intercept)	0.64	−0.10	2.32			0.021
**Step 1: Sociodemographic data**				0.046	0.046	
Age	−0.00	−0.04	−0.95			0.342
Gender: Female	0.06	0.09	1.28			0.203
Place of residence: Medium-sized city	0.09	0.12	1.75			0.082
Place of residence: Small town	0.04	0.06	0.34			0.738
Place of residence: Rural area	0.65	0.94	2.88			0.004
**Step 2: Medical data**				0.083	0.037	
Diabetes mellitus	0.02	0.03	0.45			0.654
Chronic kidney disease	−0.11	−0.16	−1.58			0.116
Coronary heart disease	0.04	0.06	0.94			0.347
BMI (kg/m^2^)	0.01	0.06	1.94			0.054
Depressive symptoms (PHQ-8)	0.00	0.02	0.71			0.481
**Step 3: eHealth data**				0.190	0.107	
Digital confidence	0.01	0.01	0.26			0.796
Internet anxiety	−0.10	−0.11	−2.70			0.007
Digital overload	0.01	0.02	0.53			0.594
**Step 4: UTAUT predictors**				0.731	0.541	
SI	0.53	0.52	10.70			<0.001
PE	0.10	0.12	1.81			0.072
EE	0.23	0.27	4.36			<0.001

Note. *N* = 310. In Steps 2, 3, and 4, only the newly included variables are presented. B = unstandardized beta. β = standardized beta. t = test statistic. R^2^ = determination coefficient. ∆R^2^ = changes in R^2^. EE = effort expectancy, PE = performance expectancy, PHQ-8 = Patient Health Questionnaire-8, SI = social influence, UTAUT = Unified Theory of Acceptance and Use of Technology.

**Table 3:** Patient reports for nutritional mHealth support. Needs and demands regarding nutritional mHealth support for atherosclerosis.

	M (SD)	N (%)
Availability		
Smartphone	4.67 (0.96)	
Tablet/iPad	2.55 (1.80)	
Computer	2.14 (1.61)	
Laptop	1.98 (1.55)	
Other	1.19 (0.96)	
Format		
App	4.57 (1.06)	
Audio or video materials	3.53 (1.50)	
Interactive training	3.40 (1.38)	
Downloadable materials	3.13 (1.42)	
Informative website	2.86 (1.75)	
Other	1.23 (0.97)	
Duration of use		
One week		9 (2.9)
One week to one month		8 (2.6)
One to three months		25 (8.1)
Permanently		268 (86.5)
Frequency of new content		
Daily		50 (16.1)
Weekly		138 (44.5)
Every other week		53 (17.1)
Monthly		26 (8.4)
All content should be available from the beginning		43 (13.9)
Session length		
1 to 10 min		46 (14.8)
10 to 20 min		124 (40.0)
20 to 30 min		121 (39.0)
30 to 45 min		16 (5.2)
>45 min		3 (1.0)
Total		310 (100.0)

## Data Availability

Anonymized data available upon reasonable request. The data are not publicly available due to privacy reasons.

## Abbreviations

BI—behavioral intention; BMI—body mass index; CVD—cardiovascular disease; EE—effort expectancy; eHealth—electronic health; LDL—low-density lipoprotein; mHealth—mobile health; PE—performance expectancy; PHQ-8—Patient Health Questionnaire-8; SI—social influence; UTAUT—Unified Theory of Acceptance and Use of Technology; VIF—variance inflation factor; WHO—World Health Organization.
